# Malthus in the Bedroom: Birth Spacing as Birth Control in Pre-Transition England

**DOI:** 10.1007/s13524-017-0556-4

**Published:** 2017-03-09

**Authors:** Francesco Cinnirella, Marc Klemp, Jacob Weisdorf

**Affiliations:** 1 Ifo Institute–Leibniz Institute for Economic Research at the University of Munich, Poschingerstr. 5, 81679 Munich, Germany; 2 Center for Economic Studies and ifo Institute (CESifo), Munich, Germany; 3 Centre for Economic Policy Research (CEPR), London, UK; 4 Centre for Competitive Advantage in the Global Economy (CAGE), Warwick, UK; 5 Department of Economics, University of Copenhagen, Copenhagen, Denmark; 6 Department of Economics, Brown University, Providence, RI USA; 7 Population Studies & Training Center, Brown University, Providence, RI USA; 8 Department of Business and Economics, University of Southern Denmark, Campusvej 55, 5230 Odense M, DK Denmark

**Keywords:** Spacing, Birth intervals, Birth control, Fertility limitation, Preventive check

## Abstract

We use duration models on a well-known historical data set of more than 15,000 families and 60,000 births in England for the period 1540–1850 to show that the sampled families adjusted the timing of their births in accordance with the economic conditions as well as their stock of dependent children. The effects were larger among the lower socioeconomic ranks. Our findings on the existence of parity-dependent as well as parity-independent birth spacing in England are consistent with the growing evidence that marital birth control was present in pre-transitional populations.

**Electronic supplementary material:**

The online version of this article (doi:10.1007/s13524-017-0556-4) contains supplementary material, which is available to authorized users.

## Introduction

The existence of marital birth control before the fertility transition of the nineteenth century is a core question among historical demographers. Early statistical analyses have shown an overall absence of marital birth control in pre-transitional England (Wilson 1984; Wrigley and Schofield 1983; Wrigley et al. 1997) and in other European countries (Henry 1961; Knodel 1987). More recent analyses using alternative methodologies and historical data from other countries and regions have found systematic evidence of parity-independent birth control both among natural-fertility populations and within populations in transition (Amialchuk and Dimitrova 2012; Anderton and Bean 1985; Bengtsson and Dribe 2006; Crafts 1989; David and Mroz 1989a, b; Dribe and Scalone 2010; Kolk 2011).

In this study, we employ a novel empirical strategy, which accounts for heterogeneity between families, on a well-known historical data set (the Cambridge Group’s family reconstitution data) to show that parity-independent and parity-dependent birth spacing were practiced among the sampled families in the three centuries that preceded England’s historical fertility transition. Previous studies, which used variation in English vital statistics (e.g., births per thousand women) from a sample of 404 parish registers (Wrigley and Schofield 1989), have found limited evidence of *preventive check* or *vice* behavior (e.g., Bailey and Chambers 1993; Crafts and Mills 2009; Kelly and Gráda 2012; Lee and Anderson 2002; Nicolini 2007). Our analysis relies on a subsample of 26 of the original 404 parishes (Wrigley et al. 1997). The advantage of using this subsample, containing the dates of more than 60,000 English births over the extraordinarily long period of 1540 to 1850, is that it concerns reconstituted families, enabling us to study patterns of births at the family level. In addition to information about the time elapsed between births within marriage, the subsample also provides individual-level data, including the order of births, the wife’s age at birth, and the husband’s profession.

Our evidence on parity-independent birth spacing is related to both of the Malthusian concepts of preventive checks and vices*.* That is, by employing a wide range of duration model specifications, we find a robust economically and statistically significant negative effect of real wages on the spacing of births among the sampled families. We also find a negative effect of real wages on the time between a woman’s 15th birthday on the one hand and her marriage and first child on the other. We do not, however, find any significant effects of real wages on the women’s stopping behavior. Our findings are consistent with previous works using short-term variation in prices and wages to document an inverse relationship between living standards and family birth spacing in other pre-transition populations (e.g., Amialchuk and Dimitrova 2012; Bengtsson and Dribe 2006; Dribe and Scalone 2010). Furthermore, we document that birth spacing in response to changes in the real wage was prevalent among low- and medium-income families but, as expected, not among families of high income. We also show that the response to real wages in terms of birth spacing increased with the number of surviving children, rejecting the notion that the delay of birth has a purely biological explanation. Our findings are robust to the introduction of potentially confounding factors, including the wife’s age at marriage during the birth interval as well as variables capturing episodes of high disease occurrences and undernourishment (such as excessive death rates and temperature variations).

More importantly, we document the existence of parity-dependent birth spacing. That is, while controlling for the wife’s age during her birth intervals, we establish that the time to the next birth increased significantly with the number of surviving children. A complicating factor when estimating the effect of parity on the spacing of births in a population concerns the potential heterogeneity between couples in their ability to conceive. More-fecund couples tend to have shorter birth intervals and are therefore, ceteris paribus, more likely to reach higher parities (Van Bavel 2004a, b; Van Bavel and Kok 2010). This heterogeneity may create compositional variation causing a selection bias: at higher parities, there is a higher representation of relatively more-fecund couples. A higher proportion of more-fecund couples at higher parities may hide the existence of parity-specific fertility behavior and result in the absence of a correlation between parity and birth intervals. Methods that do not account for family heterogeneity in fecundity, such as those relying on the use of age-specific parity progression ratios, will therefore not be able to properly identify the existence of parity-dependent birth spacing (Van Bavel 2004b). The nature and the size of our data set allow us to account for family heterogeneity and thus to focus on variation in birth intervals *within* families. Hence, by exploiting differences in spacing within the sampled families and controlling for age-related maternal infertility, we establish that the time to the next birth increased significantly and monotonically with the number of surviving children. This finding is consistent with evidence found among other populations worldwide during the initial stages of the fertility transition (e.g., Anderton and Bean 1985; David and Mroz 1989a, b; Friedlander et al. 1980; Gehrmann 2007; Van Bavel 2004a, b; Van Bavel and Kok 2004).

## Data

### The Family Reconstitution Data Set

The original data set, based on family reconstitutions of 26 English parishes, includes 80,704 families and 272,164 births. We begin by restricting the sample to births that occurred in the period 1540–1850, leaving out the thin tails (1536–1539 and 1851–1889). This restriction reduces the number of families to 80,198. Next, we require that the wife had at least two recorded births and that her age was known, between the ages of 15 and 45, at the time of her deliveries. The lower end of the age interval comes naturally: the Church of England did not allow women below the age of 15 to marry, and this is confirmed by the data. The rationale for the upper end is that the sampled women rarely conceived children after age 45 due to menopause. Moreover, the restriction mitigates the potential problem that births recorded as occurring after age 45 may be wrongly recorded.^1^ Our restrictions reduce the sample size to 18,220 families.

The sample is then further restricted to families for which wife’s age at marriage can be observed and dates of births (or baptisms) of all recorded family children exist. Prenuptially conceived children are included; however, birth intervals shorter than 40 weeks, which stem from either premature births or potential data entry errors, are excluded. These restrictions ultimately leave us with a total of 15,845 families and 62,223 birth events.^2^ This baseline sample includes 1,208 twin births, which are treated as single events.

The summary statistics are reported in Table 1. The upper part of the table reports the descriptive statistics concerning variation across births, and the lower part gives the descriptive statistics concerning family-specific variables. The mean age at marriage of wives is 24.5 years, and the mean age at the time of the first birth is 25.7 years, resulting in an average time span between marriage and first birth of about 1.2 years. The average birth interval is 924 days (circa 2.5 years), with a standard deviation of 455 days (about 1.2 years). The mean age among the sampled wives at any birth is 29.8 years, and the mean age at the time of their last birth is 35.7 years. The occupational title of the husband is known in slightly more than one-half of the sampled families, with the most common occupations being laborers, craftsmen, and husbandmen. For this subsample, the literacy status (inferred from signatures) is recorded for 40 % of all wives (and only after 1750), with 33 % of the wives signing their marriage certificate. The literacy rate among husbands, also known in 40 % of the regression sample, is 57 %. Because the literacy status of husbands and wives is highly correlated, we use only the literacy status of wives in our analyses.

**Table 1:** Summary statistics

Variable	Mean	SD	Min.	Max.	*N*
Individual					
Spacing (days)	924.5	455.25	281	4,368	62,223
Mother’s age at beginning of the interval	29.80	5.77	15.28	44.99	62,223
Birth order	3.32	2.25	1	19	62,223
Child death before next conception	0.16	0.37	0	1	62,223
Family Specific					
Mother’s age at marriage	24.46	4.81	15.00	44.49	15,845
Mother’s age at starting	25.68	4.96	15.28	44.95	15,845
Mother’s age at stopping	35.68	6.69	16.79	49.99	15,845
Protogenesic interval (years)	1.23	1.20	–0.08	11.57	15,845
Prenuptially conceived (dummy variable)	0.36	0.48	0	1	15,845
Laborers	0.19	0.40	0	1	15,845
Husbandmen	0.09	0.28	0	1	15,845
Craftsmen	0.10	0.31	0	1	15,845
Traders	0.04	0.19	0	1	15,845
Farmers	0.03	0.17	0	1	15,845
Merchants	0.07	0.26	0	1	15,845
Gentry	0.01	0.09	0	1	15,845
Occupation unknown	0.47	0.50	0	1	15,845
Mother’s literacy (dummy variable)	0.13	0.34	0	1	15,845
Mother’s literacy unknown (dummy variable)	0.60	0.49	0	1	15,845
Sibship size	5.02	2.60	2	21	15,845

*Source:* Cambridge Group family reconstitution data.

### Real Wages, Death Rates, and Temperatures

In addition to the aforementioned demographic data, our analysis also uses statistics concerning national real wages, crude death rates, and temperatures. Our key explanatory variable when testing for the existence of preventive checks (i.e., parity-independent birth spacing) is living standards measured by national real wages. The real wage series employed in the main analysis is provided by Clark (2007). The wages and prices used to compute the real wages in England are a combination of observations from across the entire country, as discussed in Clark (2007).^3^ In the duration analyses, the national yearly real wage series is combined with the demographic event data from the year in which the relevant interval started to the year in which the modeled event took place.

**Fig. 1:**
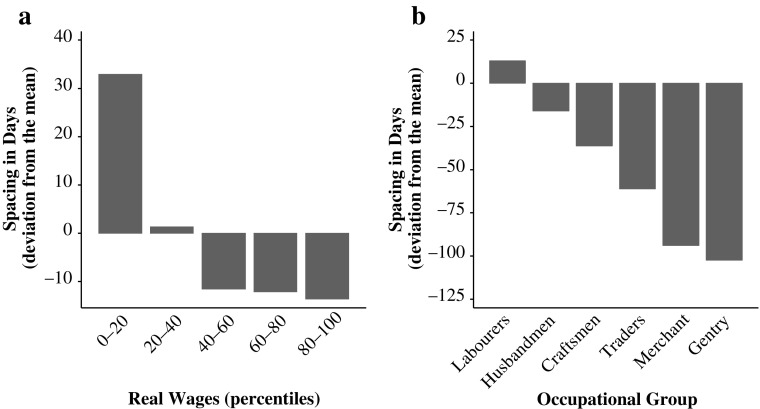
Average spacing by real-wage percentiles and occupational group

Panel a of Fig. 1 illustrates the relationship between average birth intervals and (standardized) real wages in percentiles. The graph demonstrates that periods characterized by higher real wages were associated with shorter birth intervals, suggesting a fertility response to changing economic conditions. Similarly, in illustrating average spacing of births by occupational groups, panel b of Fig. 1 shows that more-affluent social strata (traders, merchants, and gentry) had comparatively shorter birth intervals, suggesting that marital birth spacing was widespread among lower socioeconomic ranks.

Any analysis that considers birth spacing as a measure of birth control has to address a number of confounding factors that are linked to biology. The key suspects are undernourishment and climatic conditions, both of which can influence the ability to conceive (Bongaarts 1980; Lam and Miron 1996). Both of these factors are also likely to be correlated with real wages through food prices. In further robustness analyses, we use two additional series of data—one of crude death rates and one of surface air temperatures—to identify and control for harvest failure and undernourishment.

The longest historical series of surface air temperatures was recorded in England. Provided by Manley (1953) and starting in 1659, the series covers a substantial part of our period of investigation. A national series of crude death rates covering the entire period of investigation, 1540 to 1850, is available from Wrigley and Schofield (1989). The descriptive statistics of the control variables and (standardized) real wage by subperiod are reported in Table 2.

**Table 2:** Summary statistics of aggregate variables

Variable	1540–1850	1540–1600	1600–1650	1650–1700	1700–1750	1750–1800	1800–1850
	(1)	(2)	(3)	(4)	(5)	(6)	(7)
Real Wage	70.83	80.73	60.71	65.74	69.45	67.49	78.57
	(11.70)	(14.16)	(6.19)	(8.61)	(7.60)	(5.54)	(9.44)
Crude Death Rate	26.93	26.54	25.66	29.36	28.94	27.21	23.64
	(4.47)	(6.23)	(3.92)	(4.07)	(4.11)	(1.69)	(1.65)
Mean Temperature	9.04			8.64	9.26	9.07	9.10
	(0.64)			(0.62)	(0.59)	(0.57)	(0.65)

*Note:* Standard deviations are shown in parentheses.

*Source:* Real wages from Clark (2007); crude death rates from Wrigley and Schofield (1989); temperatures are from Manley (1953) and are available from 1659.

## Duration Analysis

To explore the effects of real wages and parity on fertility responses, we estimate the hazard rates of four different events: (1) marriage; (2) first conception (“starting”); (3) conception following the previous birth (“spacing”); and (4) last conception (“stopping”), for which the date of a conception is set 40 weeks prior to the date of a birth.^4^ The unit of observation in the marriage, starting, and stopping analyses is the wife. The outcome variable here measures the time span from when the wife becomes *at risk* until the relevant event occurs.^5^ In the marriage, starting, and stopping analyses, where the relevant events are the marriage, the first conception, and the last birth, respectively, the unit of observation is the family (i.e., there is one observation per family), and the family is considered to be at risk from the point in time at which the wife reaches the age of 15. In the spacing analysis, where the relevant event is the conception following the birth of the previous child, the unit of observation is the birth interval (i.e., there are potentially multiple events per family), and the wife is considered at risk of conceiving her next child at the birth of the previous child. The outcome variable is, therefore, the time span from the date of birth or baptism of one child until the date of conception of the subsequent child. The date of conception is calculated by subtracting nine months from the date of birth or baptism. In our analysis, we consider only closed birth intervals.^6^

Of note is that 90 % of our sampled birthdates are inferred from baptism dates. Previous studies have shown that most children were baptized within one month of birth (Midi Berry and Schofield 1971). Yet, a potential problem is that the time elapsed between birth and baptism may have differed systematically over time, across the sampled parishes, and across occupational groups. However, because our estimates are either based on variation within families or stratified by parish and quarter-century, such differences are accounted for.

In regressions investigating parity-independent birth spacing, each of the four events is regressed on national real wages for each of the years over the modeled interval.^7^ Furthermore, dummy variables indicating the order of surviving births (here denoted “net parity”) are included in regressions investigating parity-dependent birth spacing.^8^ We control for the income class of the husband based on his occupation; the wife’s age at marriage;^9^ the wife’s age at the beginning and during the birth interval;^10^ the wife’s literacy status;^11^ and a proxy for the couple’s fecundity (i.e., capacity to conceive) measured by the time elapsed between marriage and first birth (i.e., the *protogenesic interval*). To capture the possibly nonlinear association between fecundity and age, we include a quadratic polynomial of maternal age that varies during the birth interval. We also account, again in a time-varying fashion, for the death of the previous child before the next conception. Finally, as is common in the literature, we include a binary variable for the last birth interval to capture a failed attempt to stop having children (Anderton 1989; Knodel 1987; Okun 1995; Van Bavel 2004a).^12^

We estimate a time-varying Cox proportional hazard model (Cox 1972) specified as follows:

1 $$ h(t)={h}_o(t) \exp \left({\upbeta}_1{x}_1+{\upbeta}_2{x}_2+\cdots +{\upbeta}_k{x}_k+ g(t)\left(\upgamma W\right)\right). $$

The term *h*_*o*_(*t*) is the baseline hazard function where *t* is time, measured in days; (*x*_1_, . . . , *x*_*k*_) are socioeconomic and demographic covariates; and *W* is the standardized (zero mean and unit standard deviation) time-varying (yearly) real wage (Clark 2007). In all our analyses, we stratify by parish and quarter-century: that is, each parish and quarter-century provides unique baseline hazard functions. With this stratification, our analyses account for the heterogeneity between different time periods and locations. The stratification by quarter-century furthermore implies that the estimated impact of real wages on birth intervals can be interpreted as a short-term effect. Finally, although demographic events are recorded on specific dates, the real wages are annual averages, and so our standard errors are clustered by the year of the demographic event considered.^13^

### Parity-Independent Birth Spacing

Table 3 reports the estimates of our duration models capturing the effects of the real wage and the control variables on the duration to each of the studied events. Real wages are standardized with a mean of 0 and a standard deviation of 1. To ease comparison with previous studies, we report hazard ratios.

**Table 3:** The impact of the real wage on marriage, starting, spacing, and stopping

	Marriage	Starting	Spacing	Stopping
	(1)	(2)	(3)	(4)
Real Wage	1.235^†^	1.252*	1.097**	1.006
	(0.142)	(0.122)	(0.012)	(0.089)
Husbandmen	0.982	0.964	1.083**	1.268**
	(0.030)	(0.030)	(0.019)	(0.112)
Craftsmen	0.923**	0.924**	1.074**	1.167^†^
	(0.026)	(0.026)	(0.018)	(0.093)
Traders	0.958	0.951	1.173**	1.483**
	(0.041)	(0.034)	(0.029)	(0.164)
Farmers	0.992	0.930^†^	1.145**	1.068
	(0.046)	(0.037)	(0.027)	(0.139)
Merchants	0.995	0.962	1.190**	1.310*
	(0.041)	(0.037)	(0.024)	(0.151)
Gentry	1.133	1.087	1.189**	1.948**
	(0.098)	(0.077)	(0.056)	(0.448)
Occupation Unknown	0.911**	0.882**	1.079**	1.170*
	(0.022)	(0.022)	(0.015)	(0.084)
Mother Literacy	0.986	0.990	1.048**	1.297**
	(0.023)	(0.022)	(0.016)	(0.087)
Mother Literacy Unknown	0.896**	0.738**	0.974	1.207^†^
	(0.036)	(0.019)	(0.021)	(0.123)
Child Death			1.740**	
			(0.023)	
Mother’s Age			0.862**	0.777**
			(0.006)	(0.035)
Mother’s Age (squared)			1.002**	1.000
			(0.000)	(0.001)
Protogenesic Interval (years)			0.983**	0.989
			(0.005)	(0.019)
Prenuptially Conceived (dummy variable)			0.969**	1.022
			(0.010)	(0.052)
Last Birth Interval			0.580**	
			(0.011)	
Birth Order			1.054**	
			(0.004)	
Sibship Size				0.518**
				(0.008)
Age at Marriage Fixed Effects	No	No	Yes	Yes
Number of Subjects	19,845	22,622	62,223	3,795

*Notes:* Cox proportional hazard model with time-varying real wages is shown. Hazard ratios are reported. Real wages are standardized with zero mean and unit standard deviation. “Laborers” is the reference group. Mother’s age is measured at the beginning of the interval. Standard errors, shown in parentheses, are clustered by the year of birth. Estimates are stratified by parish and quarter-century.

^†^ *p* < .10; *p < .05; **p < .01

Column 1 of Table 3 establishes that the real wage is positively and significantly correlated with the hazard of marriage. This is prima facie evidence of a direct negative effect of living standards on the wife’s age at marriage, supporting the Malthusian hypothesis that delayed marriage was a response to hard times as well as a sign of the existence of a preventive check mechanism operating among the sampled population in pre-transitional England.

Column 2 focuses on the event of giving birth to the first child within marriage (“starting”). The estimates indicate a positive and statistically significant correlation between the real wage and the protogenesic interval. The magnitude of the effects on the events of marriage and starting are very similar: a 1 standard deviation increase in the real wage accelerates time to marriage and to first conception by 23 % and 25 %, respectively. This is consistent with the conventional view that marriage historically marked the onset of a family (i.e., to give birth).

The estimates in column 3 present evidence of pretransitional, parity-independent birth spacing, establishing that an increase in real wages accelerates the timing of the next conception. The magnitude of the impact of the real wage is also economically significant: a 1 standard deviation increase in the real wage accelerates the timing of the next conception by approximately 10 %.^14^

Turning to the stopping specification (column 4), we find no statistically significant impact of real wages on the hazard of the last conception. The effect remains statistically insignificant when we consider starting ages other than 15 for the event of stopping and when we split the sample by 50-year subperiods (not reported). These results are perhaps unsurprising: because real wages are largely nontrending across the period under observation, short-term variations in real wages are likely to cancel out over the course of a family life cycle, leaving little room for wages at any point in time to substantially affect the timing of the last birth.^15^

In summary, our analyses establish that falling living standards captured by lower real wages led not only to significantly later marriages but also to longer birth intervals within marriage. In our robustness analyses, we explore the impact of some key confounding factors to rule out the possibility that the spacing effects we observe are positive checks rather than preventive checks.

### Socioeconomic Factors and Other Covariates

To shed light on the role of socioeconomic factors in historical birth patterns, we subdivided our sampled families into income groups using a categorization proposed by Clark and Cummins (2015). Clark and Cummins used information about male testators to group male occupations according the amount of wealth left in the will. From poorest to richest, these groups are laborers, husbandmen, craftsmen, traders, farmers, merchants, and gentry. Our reference group in the analysis is laborers (the poorest group in the classification scheme). Concerning the hazard of a marriage, apart from craftsmen—who tended to marry later in life than others—none of the groups differ significantly from laborers (Table 3, column 1). When looking at the timing of the first birth, craftsmen—but also farmers—had their firstborns comparatively later in life (column 2).

More interestingly, looking at birth spacing, we find that poorer families had longer birth intervals on average than richer ones: column 3 shows that all six occupational groups included in the model have significantly shorter birth intervals (higher hazard ratios) compared with the reference group (laborers). In particular, we find that the coefficients for the richest groups (traders, farmers, merchants, and gentry) are statistically different from the coefficients of husbandmen and craftsmen; the difference between husbandmen and craftsmen is not statistically significant. Therefore, birth intervals appear to decrease with wealth.

The mechanism causing these differences in birth intervals between rich and poor may have to do with differences in breast-feeding practices. Women in poor families would breast-feed their own children, but the rich could afford to pay a wet nurse, explaining why the more-affluent social groups display a larger hazard of a further birth (Fildes 1987). Differences in the practice of *coitus interruptus* may also explain the different patterns of birth spacing (Santow 1995).

We also find a large impact of a child death on the next conception, with a child death accelerating the timing of the next conception by some 74 %. Possible reasons for this effect include the interruption of the breast-feeding period (which shortens postpartum amenorrhea) and the attempt to replace the deceased child.

Interestingly, socioeconomic differences also apply in the case of stopping. Column 4 establishes that, on average, laborers stopped later than their more-affluent counterparts and that the gentry were more likely to stop earlier. Husbandmen, craftsmen, traders, and merchants also stopped significantly earlier than laborers. These results are conditional on the mother’s age at marriage, her age at the last birth, and the family size. In fact, we find that a larger family size was associated with a later time of stopping. Therefore, differences in sterility associated with differences in the age at marriage or family size cannot explain the differences in stopping practice across occupational groups. The fact that the rich had more surviving offspring than the poor, as demonstrated by Clark and Hamilton (2006) and Boberg-Fazlic et al. (2011), can thus be ascribed to earlier starting and shorter birth intervals. The earlier stopping pattern among the rich (especially gentry) is consistent with the notion that wealthier families may have had a target number of offspring (for a discussion, see Van Bavel 2004a).

Literacy among wives is also associated with shorter birth intervals and earlier stopping age, even after we control for socioeconomic status. Perhaps literate individuals from the lower socioeconomic ranks imitate the fertility patterns of their higher socioeconomic counterparts. Moreover, couples of low fecundity, captured by a relatively large protogenesic interval, had (as expected) significantly larger birth intervals than couples of high fecundity. Also, the group of couples that gave birth to children within 40 weeks of marriage (which includes couples that conceived their firstborn before marriage) had an overall lower hazard of subsequent births. The latter finding seemingly contradicts the suggestion made by Wrigley et al. (1997:422) in their description of the data’s prevalence of prenuptially conceived births: “It might be expected that such women [giving prenuptially conceived birth] would display higher fertility during the balance of their childbearing life than women whose first child was born more than nine months after marriage, since it might be supposed that women of high fecundity, or perhaps with a greater appetite for sexual activity, would have higher fertility and would be more likely to become pregnant before marriage than others.”

Last, as documented in previous studies, we find that the last birth interval was significantly larger, on average, than the previous intervals. This finding is consistent with the idea that the last birth was sometimes a failed stopping attempt.

Before we proceed to explore the role of parity in detail, it is useful to take a preliminary look at the variable *birth order*. The coefficient for birth order (Table 3, column 3) is highly statistically significant and suggests that higher parities are associated with *shorter* spacing. As discussed in the Introduction, this finding may arise from a selection bias stemming from the use of variation in birth spacing *across* families rather than *within* them. That is, as we move from lower to higher birth orders, the composition of the sampled families may shift toward a higher share of more-fecund couples, and hence couples of shorter-than-average spacing. As the birth order results of Table 3 indicate, the composition effect may lead us to mistakenly conclude that higher parities were associated with shorter spacing of births. However, given the nature of our data, this issue of selection bias can be addressed by accounting for between-family heterogeneity. To shed light on these matters, the next section explores variation in birth spacing across families as well as within them.

### Parity-Dependent Birth Spacing

This section is devoted to the question of whether birth spacing depended on the stock of surviving offspring in a family. More specifically, we test the hypothesis that the timing of a successive birth is independent of the number of children already born (e.g., Henry 1953). We conduct the test similar to those in previous studies (e.g., Bengtsson and Dribe 2006; Van Bavel 2004a) by estimating parity fixed effects. In particular, we define net parity as the number of children alive at the start of the interval and include in the model a dummy variable for each net parity. Importantly, to test for parity-specific birth spacing appropriately, we also account for between-couple heterogeneity: that is, the fact that highly fecund couples are able to have shorter birth intervals, on average, and hence can reach higher parities, causing a potential selection bias toward shorter spacing at higher parity. We account for this selection bias by stratifying our sampled birth intervals on the family level.

Table 4 presents the estimates from duration models of birth intervals with and without stratifying on the family level. The different estimates reported in columns 1 and 2 of Table 4 illustrate the relevance of stratifying on the family level, indicating also the main reason for why our findings deviate from those of the Cambridge Group (e.g., Wrigley et al. 1997). Column 1 reports the results of using our previous spacing model augmented with only parity fixed effects. In this specification, we stratify the model by parish and quarter-century and control for the wife’s age at the beginning and during the birth interval (using a linear and a quadratic term) to capture age-related variation in maternal fecundity: we do *not* stratify by family.

**Table 4:** Parity-dependent birth spacing

	Parish Fixed Effects	Family Fixed Effects	Family Fixed Effects
	(1)	(2)	(3)
Real Wage	1.090**	1.137**	1.039^†^
	(0.008)	(0.014)	(0.023)
Net Parity 2	0.901**	0.476**	0.479**
	(0.010)	(0.010)	(0.010)
Net Parity 3	0.889**	0.276**	0.279**
	(0.012)	(0.008)	(0.009)
Net Parity 4	0.914**	0.174**	0.176**
	(0.014)	(0.007)	(0.007)
Net Parity 5	0.925**	0.109**	0.110**
	(0.016)	(0.006)	(0.006)
Net Parity 6 and Above	1.017	0.060**	0.061**
	(0.019)	(0.004)	(0.004)
Child Death	1.728**	2.839**	2.844**
	(0.024)	(0.062)	(0.062)
Last Birth Interval	0.573**	0.581**	0.578**
	(0.006)	(0.011)	(0.011)
Mother’s Age	0.894**	1.190**	1.185**
	(0.006)	(0.019)	(0.019)
Mother's Age (squared)	1.002**	0.999**	0.999**
	(0.000)	(0.000)	(0.000)
Real Wage × Net Parity 2			1.076**
			(0.025)
Real Wage × Net Parity 3			1.133**
			(0.030)
Real Wage × Net Parity 4			1.126**
			(0.033)
Real Wage × Net Parity 5			1.144**
			(0.037)
Real Wage × Net Parity 6 and Above			1.112**
			(0.039)
Number of Subjects	71,164	71,164	71,164

*Notes:* Cox proportional hazard model with time-varying real wages is shown. Hazard ratios are reported. Real wages are standardized with zero mean and unit standard deviation. Mother’s age is measured at the beginning of the interval and varies within the birth intervals. Standard errors, shown in parentheses, are clustered by household. Model in column 1 is stratified by parish. Models in columns 2 and 3 are stratified by household. All specifications are stratified also by quarter-century.

^†^ *p* < .10; **p < .01

The findings reported in column 1 of Table 4 show that the speed of a successive conception is significantly lower at parity 2 or higher compared with the reference group (parity 1). In addition, the difference between parity 2 and the remaining (higher) parities is statistically the same. This latter result is consistent with the Cambridge Group’s finding that “birth interval lengths changed very little between parities 2 and 5” (Wrigley et al. 1997:435). Our latter finding would therefore support the natural fertility hypothesis in that the spacing of births (after parity 1) does not appear to depend on parity.

**Fig. 2:**
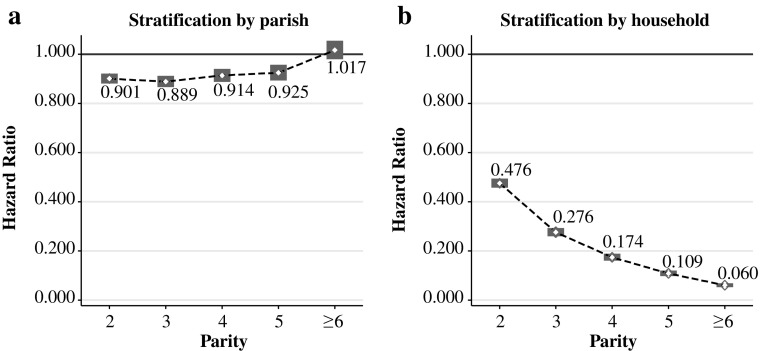
The impact of parity on birth spacing

Column 2, instead, reports the results when we account for heterogeneity among the sampled couples, stratifying by family and quarter-century. We stratify also by quarter-century to allow the baseline hazard to vary over time.^16^ By using variation in birth spacing *within* families, we find that the speed of a successive conception *decreases* monotonically with net parity, meaning that the spacing of births *increases* monotonically with net parity. For example, the coefficient for “Net parity 2” implies that the time to the successive conception after the second sibling is approximately 52 % lower than after the first sibling; the time to the next conception after the third sibling is 72 % lower compared with the interval after the first sibling; and the time to conception of a further sibling after the sixth child is 94 % lower with respect to the spacing between the first two siblings. These effects are significantly different from each other, as shown in Fig. 2, where we depict the coefficients for net parity with the relative confidence intervals estimated in columns 1 and 2 of Table 4. The figure clearly shows how *not* accounting for family heterogeneity conceals the positive impact of parity on the spacing of family births. We obtain these findings while accounting for age-related changes in maternal fecundity by controlling, in a time-varying fashion, for the age (and its square) of the mother during the interval. Interestingly, we find that after we account for family heterogeneity, the impact of child death on the successive birth interval increases in size. This finding suggests that unobserved heterogeneity at family level is correlated with child death and estimates not accounting for family heterogeneity provide biased estimates.

The impact of the real wage on the spacing of births remains highly significant also in the specifications including parity dummy variables. This leaves an important question: Does the effect of wages on births vary with parity? The underlying hypothesis here is that the decision to postpone a birth during hard times may be exacerbated by the presence of other dependent children. We test this hypothesis by interacting the real wage with the parity fixed effects. As shown in column 2 of Table 4, we stratify by family and quarter-century. Column 3 reports the results, establishing that not only are higher parities associated with significantly larger birth intervals but that the size of the demographic response to changing real wages also rises significantly with parity. The coefficients of the interactions show that the impact of real wages on spacing increases up to parity 3 (the interval between the third and fourth sibling) and then stabilizes. The coefficients for the interaction terms imply that if the real wage decreases by 1 standard deviation, the time to the next conception is approximately 8 % lower for the third child (parity 2) and 13 % lower for the fourth child (parity 3) compared with that of the second child (parity 1, the reference group).^17^ The fact that the real-wage effect varies across parity seems to suggest that birth spacing was a deliberate decision rather than a biological mechanism.^18^

It is also possible to quantify the effects of the real wage and of parities in terms of time. Consider the baseline estimate with parity fixed effects and stratification by family as in column 2 of Table 4. The birth interval associated with the first parity (first two siblings) is 493 days; the birth interval associated with parity 2 (siblings 2 and 3) is 599 days, for a difference of 106 days. This difference increases to 161 days if we consider the birth interval between siblings 3 and 4 with respect to the first interval in the family.^19^ As for the real wage, an increase of the real wage by 1.5 standard deviations is associated with the postponement of a conception by about 54 days.^20^

Note that the mechanical association between parity and the mother’s age at conception could affect the parity fixed-effect estimates. Moreover, for high parities, parity might be positively correlated with fecundity (Larsen and Vaupel 1993), which in turn would influence the length of the birth intervals affecting the size of our coefficients. To account for these factors, we reestimate in Table 5 our model with parity fixed effects for different age groups of mothers, constraining the sample to families that reach a maximum of five children. The rationale is that within a given age group, such as mothers aged 15–24, age-related fecundity is fairly constant, allowing us to estimate the “true” effect of parity on birth spacing. Moreover, by constraining the sample to families with a maximum of five children, we avoid the issue that high parities are independently correlated with fecundity and hence birth spacing.

**Table 5:** Parity-dependent birth spacing by mother’s age

	Mother’s Age 15–24	Mother’s Age 25–29	Mother’s Age 30–34	Mother’s Age 35–45
	(1)	(2)	(3)	(4)
Real Wage	1.249*	1.082	1.098	1.131
	(0.113)	(0.097)	(0.121)	(0.097)
Net Parity 2	0.411**	0.304**	0.319**	0.621**
	(0.030)	(0.030)	(0.037)	(0.064)
Net Parity 3	0.237**	0.146**	0.151**	0.496**
	(0.037)	(0.026)	(0.031)	(0.080)
Net Parity 4	0.140**	0.072**	0.055**	0.303**
	(0.081)	(0.024)	(0.022)	(0.081)
Child Death	3.977**	3.774**	3.479**	2.797**
	(0.467)	(0.468)	(0.481)	(0.350)
Last Birth Interval	0.727**	0.772*	0.745*	0.698**
	(0.074)	(0.092)	(0.103)	(0.073)
Number of Subjects	5,870	6,620	5,083	4,255

*Notes:* Cox proportional hazard model with time-varying real wages is shown. Hazard ratios are reported. Real wages are standardized with zero mean and unit standard deviation. All models are stratified by household and quarter-century. Standard errors, shown in parentheses, are clustered by household.

*p < .05; **p < .01

The estimates shown in column 1 of Table 5 support our previous findings. Within each age group, growing parities are associated with longer birth intervals. Interestingly, we also find that the impact of the real wage on birth spacing is larger and highly significant among young mothers (column 1), suggesting that households responded more strongly to changes in economic conditions during early stages of their life, when they were presumably more financially unstable.^21^

Our evidence of parity-dependent spacing is similar to that observed by Van Bavel (2004a) and others studying settings elsewhere in Europe during later periods: the larger the size of the families, the more the couples strive to postpone the next birth.

## Robustness Checks

In this section, we present analyses of the robustness of our results. Our main interest concerns fertility behavior within marriage, so here we focus exclusively on the spacing analysis outlined earlier. We conduct all our robustness checks in a model with parity fixed effects in which we stratify births by family and quarter-century.

### Subperiods

Table 6 estimates our model for the following subperiods: 1540–1599, 1600–1649, 1650–1699, 1700–1749, 1750–1799, and 1800–1850. The impact of the real wage on spacing is always statistically significant, except for the very early period, 1540–1599. The lack of significance during the sixteenth century is partly due to the lower number of observations (1,357 birth intervals), resulting in a more imprecisely estimated coefficient, as reflected by the larger standard error.

**Table 6:** The impact of real wages on spacing by subperiod

	1540–1599	1600–1649	1650–1699	1700–1749	1750–1799	1800–1849
	(1)	(2)	(3)	(4)	(5)	(6)
Real Wage	1.062	1.208**	1.095**	1.156**	1.096**	1.073**
	(0.077)	(0.047)	(0.032)	(0.030)	(0.024)	(0.029)
Net Parity 2	0.347**	0.422**	0.478**	0.485**	0.510**	0.544**
	(0.054)	(0.027)	(0.030)	(0.024)	(0.016)	(0.020)
Net Parity 3	0.182**	0.227**	0.254**	0.266**	0.310**	0.348**
	(0.046)	(0.023)	(0.025)	(0.020)	(0.014)	(0.019)
Net Parity 4	0.087**	0.133**	0.150**	0.165**	0.205**	0.229**
	(0.030)	(0.018)	(0.020)	(0.017)	(0.013)	(0.016)
Net Parity 5	0.061**	0.077**	0.096**	0.098**	0.126**	0.154**
	(0.027)	(0.013)	(0.017)	(0.013)	(0.010)	(0.014)
Net Parity 6 and Above	0.026**	0.035**	0.049**	0.052**	0.078**	0.083**
	(0.015)	(0.008)	(0.011)	(0.009)	(0.008)	(0.009)
Child Death	5.366**	3.869**	3.423**	2.937**	2.400**	2.476**
	(0.944)	(0.265)	(0.235)	(0.146)	(0.082)	(0.105)
Last Birth Interval	0.698*	0.598**	0.538**	0.536**	0.564**	0.610**
	(0.101)	(0.035)	(0.034)	(0.025)	(0.017)	(0.019)
Mother’s Age	1.508**	1.150**	1.113*	1.081*	1.133**	1.216**
	(0.175)	(0.056)	(0.058)	(0.042)	(0.027)	(0.032)
Mother’s Age (squared)	0.996*	1.000	1.001	1.001	1.000	0.999**
	(0.002)	(0.001)	(0.001)	(0.001)	(0.000)	(0.000)
Number of Subjects	1,357	6,793	6,184	11,708	25,340	19,731

*Notes:* Cox proportional hazard model with time-varying real wages is shown. Hazard ratios are reported. Real wages are standardized with zero mean and unit standard deviation. Mother’s age is measured at the beginning of the interval and varies within the birth intervals. All models are stratified by household and quarter-century. Standard errors, shown in parentheses, are clustered by household.

*p < .05; **p < .01

The analysis by subperiods suggests that the response in spacing to changes in real wages was most pronounced in the periods 1600–1649 and 1700–1749. This latter finding is consistent with the findings of Kelly and Gráda (2012), who observed a rising impact of wages on birth rates in the early eighteenth century. The point estimate for the subperiod during which we have the strongest preventive check (1600–1649) reveals that a 1 standard deviation increase in the real wage was associated with a 21 % increase in the speed of a successive conception. Again, the coefficients for the parity fixed effects confirm the existence of parity-specific spacing during each of our subperiods.

### Compositional Effects

Because data are unavailable for some parishes for some periods, the composition of parishes in our analysis changes over time. Likewise, the occupational titles of husbands are more frequently reported toward the end of the period under consideration. To the extent that the likelihood of inclusion in the regression sample is correlated with spacing behavior, the estimated relationships may be biased. Although the stratification by quarter-century, parish, or family already accounts for the potential confounding effects of time, location, or family, respectively, we assess the influence of sample selection by focusing on parishes without attrition, and with full occupational coverage. As a further assessment, we investigate the robustness of our results when we control for time fixed effects on a higher resolution than quarter-centuries.^22^

The results are reported in Table S3 in Online Resource 1. In column 1, we estimate our model using a subsample containing those 12 parishes with continual coverage across the period 1600–1800.^23^ The impact of the real wage on birth intervals remains highly significant and of similar size with respect to the baseline estimate reported in the previous section. In column 2, we include only families in which the husband has an occupational title recorded: our findings are robust to this subsample, also.^24^

Along similar lines, although the stratification of birth intervals by quarter-century accounts for changes in quarter-century fixed effects, it does not account for potential secular changes affecting both the real wages and the demographic outcomes on a shorter timescale. Column 3 establishes that when accounting for decade fixed effects instead of quarter-century fixed effects (while still stratifying by family), the estimated impact of the real wage on birth intervals remains highly significant both statistically and economically. Thus, the estimated effect of aggregate wages on the hazard of births within families cannot be attributed to secular variations across quarter-centuries. Overall, these specifications indicate that the qualitative results cannot be attributed to sample selection bias.

### Migration

Another limitation of the data is that the migration of people in or out of the sampled parishes is not detected, which presents a problem if the decision to migrate is correlated with both real wages and actual birth intervals, or if migrants and nonmigrants have different spacing behavior. Although there are, of course, limits to what we can do to deal with the problem of migration, we address the issues in two ways: we account for *permanent* migration by including a dummy variable indicating those husbands and wives who had a missing birth or death date, and we account for *temporary* migration by excluding birth intervals lengthy enough to potentially conceal unobserved births. Furthermore, we also follow Ruggles (1999) and restrict our sample to so-called completed marriages, ensuring that the sampled husbands and wives did not terminate the marriage prematurely by migration or death.

To investigate whether movers are different from stayers in terms of spacing behavior, we first exclude immigrants and then emigrants from the baseline sample. We define a couple as an immigrant couple (i.e., coming from an unobserved parish) if the husband and wife both have missing birth/baptism dates but recorded death/burial dates. Furthermore, we define a couple as an emigrant couple (i.e., moving to an unobserved parish) if the husband and wife both have missing death/burial dates but recorded birth/baptism dates. Table 7 shows that it makes virtually no difference to the effect of real wages on spacing whether we exclude immigrant couples (column 1) or emigrant couples (column 2) compared with the baseline estimate (Table 3, column 3). Including dummy variables indicating immigrants and emigrants (not shown) generates similar results.

**Table 7:** Accounting for migration

	Immigrants	Emigrants	Spacing < 3 Years	Spacing < 2.5 Years	Completed Marriages
	(1)	(2)	(3)	(4)	(5)
Real Wage	1.086**	1.099**	1.110**	1.080**	1.144**
	(0.018)	(0.035)	(0.016)	(0.019)	(0.024)
Net Parity 2	0.684**	0.500**	0.531**	0.544**	0.509**
	(0.018)	(0.024)	(0.012)	(0.014)	(0.019)
Net Parity 3	0.534**	0.299**	0.374**	0.413**	0.299**
	(0.017)	(0.022)	(0.013)	(0.016)	(0.016)
Net Parity 4	0.447**	0.187**	0.283**	0.322**	0.194**
	(0.018)	(0.019)	(0.013)	(0.016)	(0.014)
Net Parity 5	0.382**	0.126**	0.210**	0.248**	0.125**
	(0.020)	(0.016)	(0.012)	(0.016)	(0.011)
Net Parity 6 and Above	0.307**	0.064**	0.157**	0.208**	0.076**
	(0.019)	(0.010)	(0.011)	(0.017)	(0.009)
Child Death	2.658**	2.849**	3.024**	2.988**	2.729**
	(0.091)	(0.161)	(0.076)	(0.083)	(0.105)
Last Birth Interval	0.733**	0.601**	0.796**	0.850**	0.502**
	(0.021)	(0.026)	(0.018)	(0.024)	(0.020)
Mother’s Age		1.261**	1.103**	1.072**	1.118**
		(0.048)	(0.021)	(0.023)	(0.033)
Mother’s Age (squared)		0.998**	0.999^†^	1.000	1.000
		(0.001)	(0.000)	(0.000)	(0.000)
Number of Subjects	25,641	12,683	54,831	43,657	19,624

*Notes:* Cox proportional hazard model with time-varying real wages is shown. Hazard ratios are reported. Real wages are standardized with zero mean and unit standard deviation. Mother’s age is measured at the beginning of the interval and varies within the birth intervals. Standard errors, shown in parentheses, are clustered by household. Estimates are stratified by household and quarter-century.

^†^ *p* < .10; **p < .01

Low real wages may induce couples to temporarily leave their parish of residence in search of work. If they give birth and baptize a child while living outside their home-parish, these births will go unobserved in our data and instead appear as an extended birth spacing interval. We address this issue by excluding intervals that are comparatively long. In column 3 of Table 7, we restrict the sample to birth intervals of less than three years, roughly making up the 75th percentile of the sampled intervals. The coefficient on real wages remains highly significant and of the same order of magnitude as the baseline estimate. Column 4 shows the results for an even more restrictive assumption: namely, focusing on birth intervals of less than 2.5 years, which is close to the average length of birth intervals. Once more, we find a significant effect of real wages on spacing.

Ruggles (1999) pointed out that restricting the sample to completed marriages— those marriages in which both spouses survive to the time where the wife reaches age 50—is particularly useful to deal with issues of migration. Not only do completed marriages ensure that the sampled couples are neither permanent immigrants nor permanent emigrants—because we require their birth and death dates to be known—but they also warrant that the couples are healthy enough to not end reproduction prematurely. Column 5 of Table 7 reports the estimates based on the subsample of completed marriages: the coefficient for the real wage is still highly significant and even larger in magnitude than the baseline estimate.^25^

### Biological Influences

Is what we observe a biological mechanism rather than deliberate spacing behavior? Two potentially confounding biological factors are undernourishment and climatic conditions, as measured by air temperatures, both of which have been shown to impact fertility (Bongaarts 1980; Lam and Miron 1996). Because both of these factors are also likely to be correlated with real wages (temperatures through crop yields and hence food prices, and undernourishment when real wages are close to subsistence), we need to account for such potentially confounding mechanisms. Although we already established in the baseline analysis that the impact of real wages on spacing is parity-specific, we further address the question of biological influences by accounting for the potential confounding effects of climate and undernourishment.

Accounting for the potential confounding effect of air temperatures (provided by Manley 1953), we find that they are not significantly associated with birth intervals (Table S6, column 1, Online Resource 1). Reassuringly, the effect of the real wage and parity on birth spacing remains highly significant.

To account for the potential biological effects of unobserved correlates of real wages, such as undernourishment, we control for aggregate crude death rates (provided by Wrigley and Schofield 1989). Death rates not only reflect the disease environment—and thus, disease-related infertility—but can also capture episodes of crop failure and famines, and the effect thereof on fecundity. We find that higher death rates increase the time to the next conception (Table S6, column 2, Online Resource 1). However, the effect of the real wage on birth spacing remains highly significant. We reach the same conclusions when including both temperatures and death rates in the model (Table S6, column 3, Online Resource 1).

### Occupational Group

Our final robustness check concerns the extent to which the real-wage impact on the spacing of births differs across different occupational groups. To this end, we estimate the spacing model separately for each of the socioeconomic groups as categorized by Clark and Cummins (2015): laborers, husbandmen, craftsmen, traders, farmers, merchants, and gentry. Table 8 shows that the impact of the real wage on spacing is large and highly significant for all occupational groups except for farmers and for the joint category of merchants and gentry, indicating a significant insensitivity to real-wage variation among the wealthier sections of society.^26^ These differences in birth spacing among occupational groups are consistent with the findings of Bengtsson and Dribe (2006), observing a response in spacing to food price variations among the landless and semi-landless, but not among noble tenants and freeholders in southern Sweden. This result is also in line with the findings of Kelly and Gráda (2012), who concluded that higher wheat prices deterred marriages of less wealthy tenants, whereas they had a positive impact on wealthier families. Our findings show that although parity-independent birth spacing (the real-wage effect) pertains only to less-affluent sections of society, parity-dependent birth-spacing effects were common across the entire socioeconomic spectrum.

**Table 8:** The impact of the real wage on spacing by occupational group

	Laborers	Husbandmen	Craftsmen	Traders	Farmers	Merchants and Gentry
	(1)	(2)	(3)	(4)	(5)	(6)
Real Wage	1.122**	1.131**	1.249**	1.132*	1.103	1.060
	(0.031)	(0.046)	(0.047)	(0.060)	(0.072)	(0.046)
Net Parity 2	0.516**	0.503**	0.467**	0.520**	0.508**	0.569**
	(0.023)	(0.035)	(0.027)	(0.045)	(0.057)	(0.037)
Net Parity 3	0.325**	0.332**	0.256**	0.281**	0.342**	0.332**
	(0.022)	(0.035)	(0.022)	(0.035)	(0.053)	(0.032)
Net Parity 4	0.200**	0.207**	0.185**	0.258**	0.228**	0.211**
	(0.018)	(0.029)	(0.021)	(0.040)	(0.048)	(0.028)
Net Parity 5	0.127**	0.126**	0.126**	0.145**	0.164**	0.152**
	(0.015)	(0.023)	(0.018)	(0.029)	(0.041)	(0.024)
Net Parity 6 and Above	0.076**	0.076**	0.070**	0.086**	0.101**	0.087**
	(0.011)	(0.017)	(0.013)	(0.022)	(0.033)	(0.017)
Child Death	2.701**	2.723**	2.579**	2.283**	3.121**	2.548**
	(0.134)	(0.192)	(0.155)	(0.186)	(0.405)	(0.189)
Last Birth Interval	0.572**	0.614**	0.549**	0.558**	0.475**	0.647**
	(0.024)	(0.039)	(0.030)	(0.047)	(0.052)	(0.040)
Mother’s Age	1.216**	1.153**	1.144**	1.221**	1.194^†^	1.104^†^
	(0.041)	(0.060)	(0.051)	(0.090)	(0.111)	(0.059)
Mother’s Age (squared)	0.999*	0.999	1.000	0.999	0.999	1.000
	(0.000)	(0.001)	(0.001)	(0.001)	(0.001)	(0.001)
Number of Subjects	14,648	6,493	8,046	3,310	2,355	5,911

*Notes:* Cox proportional hazard model with time-varying real wages is shown. Hazard ratios are reported. Real wages are standardized with zero mean and unit standard deviation. Mother’s age is measured at the beginning of the interval and varies within the birth intervals. Standard errors, shown in parentheses, are clustered by household. Estimates are stratified by household and quarter-century.

^†^ *p* < .10; *p < .05; **p < .01

## Conclusion

We set out to reinvestigate the hypothesis that marital birth control in pre-transitional England was absent. To analyze this issue, we use a variety of specifications of duration models on a well-known historical data set with the timing of births as the main outcome variable. We find a large and robust effect of real wages on birth spacing consistent with previous findings on the existence of parity-independent birth control in pre-transition populations. By exploiting variation in birth intervals *within* families, which allows us to account for family heterogeneity, we establish the existence also of parity-dependent birth spacing in the sampled population in the three centuries preceding England’s fertility transition. Evidence of parity-dependent spacing holds across occupational groups and across centuries. Although changes in nutrition, health, and libido cannot be excluded as potential mechanisms, our findings are overall consistent with the hypothesis that couples adjusted the timing of their births in accordance not only with the prevalent economic conditions but also with their stock of dependent children.

## Electronic supplementary material

ESM 1 (PDF 303 kb)

## Footnotes

1 Relaxing either of these two restrictions has no influence on the qualitative nature of our results.

2 In the analyses stratified by family, the data requirements are less strict because time-invariant variables drop out. Thus, we can exploit a total of 71,164 birth events.

3 The same real-wage series was used in Kelly and Gráda (2012).

4 Because the date of conception can potentially be influenced by parents—whereas the interval between conception and birth is presumably outside parental control—the analyses of birth events focuses on dates of conceptions rather than dates of births.

5 In the marriage analysis, we focus only on first marriages of both husband and wife, and we exclude the cases for which we are sure that it is at least a second marriage. We include only individuals who eventually marry, consistent with the idea of studying the timing of marriage in response to changing economic conditions.

6 Our results are qualitatively unaffected if we allow for censoring—that is, if we consider the last birth interval as open (see Online Resource 1, Table S5).

7 For example, if a child was born on October 21, 1750, and the successive child was conceived on July 5, 1753, then the relevant real wages for this birth interval are those recorded in the years 1750, 1751, 1752, and 1753.

8 Net parity is computed as the number of children alive at the start of the interval. See Van Bavel (2004a) for a discussion on crude parity and net parity.

9 Information about the wife’s age at marriage is missing in approximately 75 % of all families. We thus include a binary variable for unknown age at marriage of the wife in the regression.

10 The age of the mother is used, similarly to the real wage, in a time-varying fashion: that is, we let the variable to increase year by year between two births.

11 Because maternal and paternal literacy are highly correlated, we include only the wife’s literacy.

12 The binary variable for last birth interval is based on the last birth recorded. Thus, we cannot rule out that the wife migrated to another parish and that she had additional births there. However, in Table 7, we address directly the issue of migration.

13 Whenever we include parity fixed effects, we cluster the standard errors by family.

14 If we exclude childbirths coming from second or higher-order marriages, we obtain virtually the same results.

15 For a methodological discussion on the relationship between spacing and stopping, see Anderton (1989) and McDonald and Knodel (1989).

16 Stratifying by quarter-century is not necessary to obtain the new results on parity. As we show later herein as well as in Online Resource 1 in Table S3, the results on parity holds also when analysing subperiods and including decade fixed effects.

17 The coefficients of the interaction terms are not statistically different from each other. However, interacting the real wage with the variable *birth order* provides a significant and positive coefficient, indicating that the effect of the real wage on spacing changes across parities.

18 We performed a standard test of the proportionality assumption based on the Schoenfeld residuals (see Online Resource 1). This test cannot reject the null hypothesis that the real wage satisfies the proportionality assumption both in the parity-independent and in the parity-dependent analysis. The proportionality assumption also cannot be rejected for the parity fixed effects.

19 These are median survival times predicted for a birth interval starting in the year 1710, from a woman aged 30 at the beginning of the birth interval, with the standardized real wage at 0. To compute the predicted median survival time, we first compute the adjusted survival probabilities for each mother at every observation (i.e., once per year of each birth interval) by raising the estimated stratum-specific baseline survival functions to the power of the linear prediction ${x}_i\widehat{\upbeta}$. We then compute the average of the survival function approximately 0.5 within the interval 0.475–0.525.

20 These predicted median survival times are adjusted for a fourth surviving child, born in 1710, from a mother who was 30 at the beginning of the birth interval.

21 If we combine all age groups of columns 2–4, the coefficient for real wages becomes significant.

22 In Online Resource 1, we show that the geographical coverage of the original sample and of the regression sample does not deviate systematically (see Figs. S1 and S2).

23 The 12 parishes are Aldenham, Banbury, Birstall, Bottesford, Colyton, Gainsborough, Gedling, Methley, Odiham, Shepshed, Southill, and Terling. The three parishes with the longest birth intervals are Terling, Dawlish, and Hartland. The three parishes with the shortest birth intervals are Lowestoft, Gainsborough, and March.

24 We also estimated a model for a subsample including the three parishes that have the richest occupational information (Austrey, Earsdon, and Gainsborough). The results are virtually identical. Moreover, Wrigley et al. (1997:43–44) suggested that the parish of Birstall may be problematic because of its large population size and the fact that its occupational structure is strongly biased toward manufacturing. Our results are, however, unaffected if we exclude Birstall.

25 In Online Resource 1, we report additional robustness checks. In Table S1, we show estimates using alternative real wage series. In Table S4, we estimate our parity-fixed effect model constraining the sample on known marriage date and on households for which the original source explicitly mentions it is a first marriage. In Table S5, we estimate our model including the last birth interval, accounting for censoring. In all cases, the results are virtually unaffected.

26 We merged the merchants and gentry groups because of the low number of observations.
