# Supplementary material for: Malthus in the Bedroom: Birth Spacing as Birth Control in Pre-Transition England
Source: Demography. 2017 Mar 9;54(2):413–36. doi: 10.1007/s13524-017-0556-4 (PMC5477677; doi:10.1007/s13524-017-0556-4)

Online Resource 1

Alternative wage series

Our baseline analysis uses real wages of farm workers from Clark (2007). Table S1 reports the estimates of our model using alternative real wage series collected by Allen, including the real wages of craftsmen from London and Oxford (columns 2 and 3) as well as the real wages of labourers from London (column 4). For ease of comparison, column 1 reports the baseline estimates using Clark's real wages. Table S1 establishes that our results are robust to the use of different real-wage series: in all our specifications the coefficients for the real wage are not only highly significant but also of similar magnitude.

Table S1: Alternative real wage series

	Baseline	Alternative real wages		
	(1)	(2)	(3)	(4)
Real wage	1.137***			
	(0.014)			
Craftsmen real wage (London)		1.118***		
		(0.021)		
Craftsmen real wage (Oxford)			1.175***	
			(0.031)	
Labourers real wage (London)				1.168***
				(0.026)
Net parity 2	0.476***	0.475***	0.475***	0.475***
	(0.010)	(0.010)	(0.010)	(0.010)
Net parity 3	0.276***	0.275***	0.275***	0.275***
	(0.008)	(0.008)	(0.008)	(0.008)
Net parity 4	0.174***	0.173***	0.174***	0.173***
	(0.007)	(0.007)	(0.007)	(0.007)
Net parity 5	0.109***	0.108***	0.108***	0.108***
	(0.006)	(0.006)	(0.006)	(0.006)
Net parity 6	0.060***	0.060***	0.060***	0.060***
	(0.004)	(0.004)	(0.004)	(0.004)
Control variables	Yes	Yes	Yes	Yes
Subjects	71164	71164	71164	71164

Note: Cox proportional hazard model with time-varying real wages. Hazard ratios reported. The real wage series in columns 2-4 are from Robert Allen and are standardized with zero mean and unit standard deviation. Control variables are: child death, last birth interval, quadratic polynomial of mother age which varies within the birth intervals, dummy variables for children born on January 1st, January 11th, and December 25th. Standard errors in parenthesis are clustered by household. Estimates are stratified by household and quarter century. * p< 0.1, ** p < 0.05, *** p < 0.01.

Representativeness of the regression sample

Table S2 reports a cross-tabulation of the parishes and occupational groups included in our sample. There is clearly a large variation across the sampled parishes, both concerning occupational structures and missing information on occupations. In parishes like Austrey and Gainsborough the

share of husbands with a recorded occupation was 80%, while Hartland and Methley had no occupational information recorded at all. Also, the practice of recording occupations becomes more common with time.

Table S2: Parishes and occupation

Parish/Class	Labourers	Husbandmen	Craftsmen	Traders	Farmers	Merchants	Gentry	Unknown
Alcester	6%	2%	7%	4%	0%	2%	6%	74%
Aldenham	13%	5%	3%	2%	4%	1%	2%	70%
Ash	26%	5%	6%	3%	9%	1%	1%	48%
Austrey	25%	18%	12%	4%	15%	3%	2%	20%
Banbury	20%	12%	22%	9%	2%	7%	1%	27%
Birstall	2%	3%	4%	1%	2%	17%	1%	69%
Bottesford	19%	12%	9%	6%	9%	2%	0%	43%
Bridford	11%	8%	4%	1%	9%	1%	0%	66%
Colyton	6%	8%	10%	3%	3%	2%	2%	66%
Dawlish	17%	5%	8%	1%	3%	6%	2%	58%
Earsdon	19%	37%	17%	4%	4%	4%	1%	14%
Gainsborough	22%	13%	22%	12%	2%	8%	1%	20%
Gedling	1%	1%	6%	0%	0%	1%	0%	92%
Great Oakley	14%	10%	6%	3%	10%	1%	0%	56%
Hartland	0%	0%	0%	0%	0%	0%	0%	100%
Ipplepen	22%	14%	10%	2%	7%	2%	2%	42%
Lowestoft	9%	14%	7%	3%	1%	9%	1%	56%
March	4%	2%	1%	1%	1%	0%	1%	90%
Methley	0%	0%	0%	0%	0%	0%	0%	100%
Morchard Bishop	18%	7%	6%	2%	7%	1%	0%	59%
Odiham	27%	7%	10%	6%	7%	5%	3%	35%
Reigate	12%	10%	14%	12%	5%	5%	4%	39%
Shepshed	37%	9%	9%	3%	3%	1%	0%	38%
Southill	6%	2%	1%	2%	2%	1%	1%	86%
Terling	32%	8%	12%	7%	3%	3%	2%	33%
Willingham	7%	1%	1%	0%	0%	1%	1%	89%
Total	15%	8%	10%	4%	3%	5%	1%	53%

Source: Cambridge Group family reconstitution data

Figure S1 shows the distribution of births by parish in the original and in the regression sample. The graph indicates that there is no major deviation in the geographical coverage when going from the original to the constrained sample. Here is the list of 26 parishes: 1 Alcester, 2 Aldenham, 3 Ash, 4 Austrey, 5 Banbury, 6 Birstall, 7 Bottesford, 8 Bridford, 9 Colyton, 10 Dawlish, 11 Earsdon, 12 Gainsbro, 13 Gedling, 14 Great Oakley, 15 Hartland, 16 Ipplepen, 17 Lowestoft, 18 March, 19 Methley, 20 Morchard Bishop, 21 Odiham, 22 Reigate, 23 Shepshed, 24 Southill, 25 Terling, 26 Willingham.

Figure S1: The distribution of births by parish in the original sample (blue) and regression sample (red)

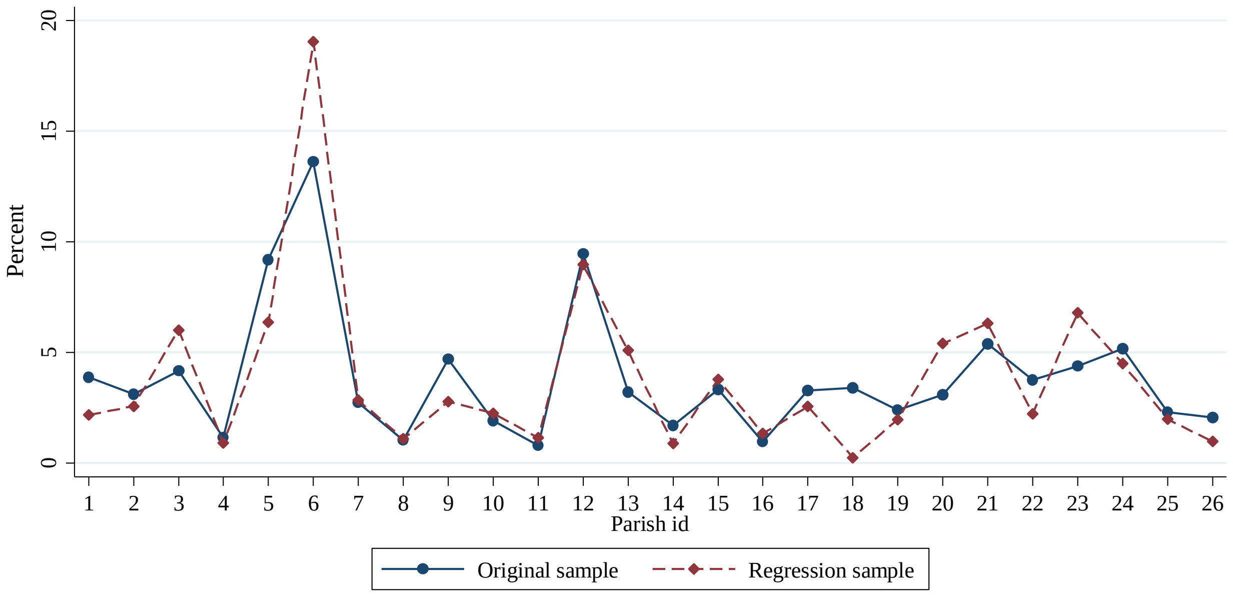

Figure S2 shows how the shares of occupational groups are distributed by parish in the original and in the regression sample. As one can see the occupational patterns of the original sample are pretty well preserved in the regression sample. The legend for the occupational groups is reported here: Occup0 = Unknown occupation; Occup1 = Labourers; Occup2 = Husbandmen; Occup3 = Craftsmen; Occup4 = Traders; Occup5 = Farmers; Occup6 = Merchants; Occup7 = Gentry; The subscript "_rs" indicates the variables used in the regression sample.

Figure S2: The shares of each occupational group by parish in the original sample (blue) and in the constrained sample (red)

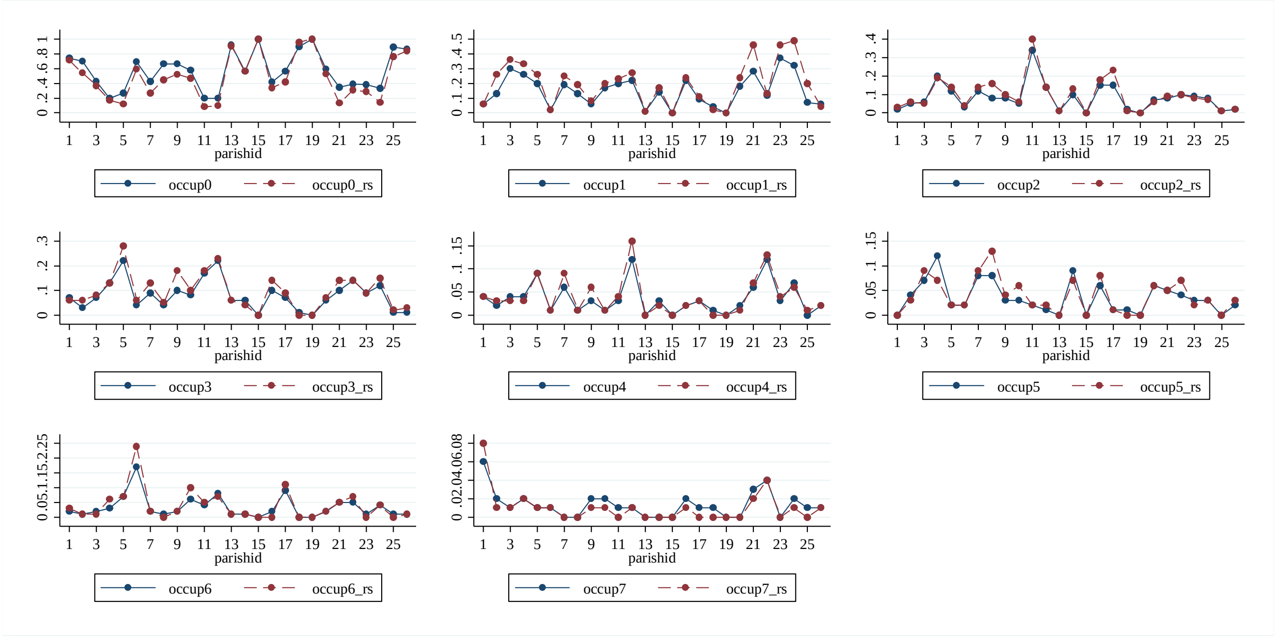

Table S3: Accounting for compositional effects

	W/o parish attrition (1)	Known occupation (2)	Decade FE (3)
Real wage	1.148***	1.141***	1.115***
	(0.017)	(0.018)	(0.013)
Net parity 2	0.474***	0.512***	0.516***
	(0.012)	(0.013)	(0.010)
Net parity 3	0.274***	0.310***	0.313***
	(0.010)	(0.012)	(0.009)
Net parity 4	0.178***	0.206***	0.204***
	(0.009)	(0.011)	(0.008)
Net parity 5	0.114***	0.135***	0.132***
	(0.007)	(0.009)	(0.006)
Net parity 6	0.065***	0.079***	0.073***
	(0.005)	(0.007)	(0.004)
Child death	2.866***	2.613***	2.658***
	(0.075)	(0.073)	(0.054)
Last birth interval	0.590***	0.578***	0.575***
	(0.013)	(0.014)	(0.010)
Mother's age	1.171***	1.176***	1.140***
	(0.022)	(0.024)	(0.016)
Mother's age (squared)	0.999**	0.999***	1.000
	(0.000)	(0.000)	(0.000)
Decade FE	No	No	Yes
Subjects	47514	40763	71164

Note: Cox proportional hazard model with time-varying real wages. Hazard ratios reported. Real wages are standardized with zero mean and unit standard deviation. Mother’s age is measured at the beginning of the interval and varies within the birth intervals. Standard errors in parenthesis are clustered by household. Estimates are stratified by household and quarter century in column 1 and 2; estimates in column 3 are stratified by household.
* p< 0.1, ** p < 0.05, *** p < 0.01.

Additional robustness checks

In the parity-dependent analysis with stratification by household we do not impose the constraint regarding knowledge about the date of marriage, and, therefore, we do not account for pre-nuptial conception. That explains why the number of observations increases from 62,223 (Table 3, column 3) to 71,164 (Table 4). In column 1 of Table S4 we constrain the sample to households with a known marriage date. The impact of the real wage and of parity is unaffected. In column 2 we show estimates restricting the sample to households for which the original source explicitly mentions that it is a first marriage. Also in this case we obtain virtually the same results although we lose about half of the observations.

Table S4: Constraining on known marriage date

	Known marriage date (1)	First marriages (2)
Real wage	1.136***	1.148***
	(0.014)	(0.019)
Net parity 2	0.474***	0.481***
	(0.010)	(0.014)
Net parity 3	0.274***	0.269***
	(0.009)	(0.012)

Net parity 4	0.174***	0.169***
	(0.008)	(0.010)
Net parity 5	0.110***	0.104***
	(0.006)	(0.008)
Net parity 6	0.061***	0.057***
	(0.004)	(0.005)
Control variables	Yes	Yes
Subjects	62223	36000

Note: Cox proportional hazard model with time-varying real wages. Hazard ratios reported. Real wages are standardized with zero mean and unit standard deviation. Control variables are: child death, last birth interval, quadratic polynomial of mother age which varies within the birth intervals, dummy variables for children born on January 1st, January 11th, and December 25th. Standard errors in parenthesis are clustered by household. Estimates are stratified by household and quarter century. * p< 0.1, ** p < 0.05, *** p < 0.01.

Table S5 shows estimates using a standard Cox model (without time-varying covariates) including the last interval. In particular, we consider an open interval, *i.e.* we censor the last birth, if we lose track of the mother (column 1). Through censoring we account for the possibility that the mother produced another birth in another parish. As one can see, both the impact of the real wage and of parity does not depend on how we treat the final open birth interval. In Table S6 we control for temperatures and crude death rates to account for potentially confounding biological effect. See discussion in the main text in section 4.

Table S5: Last birth interval (censoring)

	Open interval<br>(1)	Closed interval<br>(2)
Real wage	1.093***	1.078***
	(0.012)	(0.013)
Net parity 2	0.723***	1.262***
	(0.013)	(0.026)
Net parity 3	0.702***	1.947***
	(0.018)	(0.062)
Net parity 4	0.737***	3.158***
	(0.025)	(0.134)
Net parity 5	0.751***	4.626***
	(0.032)	(0.245)
Net parity 6	0.747***	8.610***
	(0.040)	(0.562)
Last birth interval		0.978
		(0.018)
Control variables	Yes	Yes
Subjects	89163	71073

Note: Cox proportional hazard model. Hazard ratios reported. Real wages are standardized with zero mean and unit standard deviation. Control variables are: child death, quadratic polynomial of mother's age which varies within the birth intervals, dummy variables for children born on January 1st, January 11th, and December 25th. Standard errors in parenthesis are clustered by household. Estimates are stratified by household and quarter century. * p< 0.1, ** p < 0.05, *** p < 0.01.

Table S6: Accounting for contemporary temperatures and crude death rates

	(1)	(2)	(3)
Real wage	1.128***	1.116***	1.119***
	(0.015)	(0.015)	(0.015)
Net parity 2	0.488***	0.487***	0.487***
	(0.010)	(0.010)	(0.010)
Net parity 3	0.287***	0.287***	0.287***
	(0.009)	(0.009)	(0.009)
Net parity 4	0.184***	0.183***	0.183***
	(0.008)	(0.008)	(0.008)
Net parity 5	0.115***	0.115***	0.115***
	(0.006)	(0.006)	(0.006)
Net parity 6	0.066***	0.066***	0.066***
	(0.005)	(0.005)	(0.005)
Temperature	0.993		0.993
	(0.011)		(0.011)
Crude death rate		0.992***	0.992***
		(0.003)	(0.003)
Control variables	Yes	Yes	Yes
Subjects	62327	62327	62327

Note: Cox proportional hazard model with time-varying real wages, temperatures, and crude death rates. Hazard ratios reported. Real wages are standardized with zero mean and unit standard deviation. Control variables are: child death, quadratic polynomial of mother's age which varies within the birth intervals, dummy variables for children born on January 1st, January 11th, and December 25th. Standard errors in parenthesis are clustered by household. Estimates are stratified by household and quarter century. * p< 0.1, ** p < 0.05, *** p < 0.01.

Test of the proportional-hazard assumption

The formal test based on the Schoenfeld residuals can never reject the proportionality assumption for our variable of interest, the real wage. A high *p-value* indicates a non-rejection of the proportionality assumption. The *p-value* for the real wage based on the specification with stratification by household in Table 4 (column 2) is 0.62. The test for proportionality is more meaningful if we restrict the analysis by sub-period (as in Table 6) to account for secular changes in spacing behaviour which could affect the results of the test. In this case the *p-values* for the real wage are reported in Table S7 below. The impact of the real wage on spacing based on the Cox proportional hazard model satisfies the proportionality assumption.

Table S7: Testing the proportional-hazards assumption

	Period					
	1540-1600 (1)	1600-1650 (2)	1650-1700 (3)	1700-1750 (4)	1750-1800 (5)	1800-1850 (6)
Real wage (*p-value*)	0.285	0.674	0.750	0.674	0.428	0.852

Note: Test of the proportional-hazard assumption based on the Schoenfeld residuals. The test is conducted by subperiod. *P-values* reported.

In Table S8 we report the results of the formal test using the Schoenfeld residuals for the parity-fixed effects and the other control variables. Since the dummies for the parity effects do not vary over time within birth intervals, the test is based on a standard Cox proportional hazard model (without time-varying covariates). To account for secular trends we base the test on the estimates by sub-period as in Table 6. The proportionality assumption cannot be rejected in the vast majority of the cases. Only for the variable child death and from 1700 onwards the proportionality assumption is rejected.

Table S8: Testing the proportionality assumption

	Period					
	1540-1600	1600-1650	1650-1700	1700-1750	1750-1800	1800-1850
	(1)	(2)	(3)	(4)	(5)	(6)
Real wage	0.395	0.722	0.967	0.466	0.946	0.627
Net parity 2	0.969	0.793	0.829	0.188	0.199	0.086
Net parity 3	0.742	0.896	0.790	0.273	0.481	0.172
Net parity 4	0.415	0.667	0.905	0.119	0.659	0.221
Net parity 5	0.405	0.760	0.832	0.304	0.825	0.492
Net parity 6	0.633	0.615	0.561	0.262	0.692	0.674
Child death	0.239	0.138	0.229	0.072	0.000	0.001
Last spacing	0.848	0.391	0.291	0.888	0.737	0.911
Mother's age	0.812	0.929	0.685	0.692	0.987	0.721
Mother's age sq.	0.962	0.876	0.575	0.519	0.800	0.676

Note: Test of the proportional-hazard assumption based on the Schoenfeld residuals. The test is conducted by subperiod. P-values reported.

The non-rejection of the proportionality assumption for the real wage and the parity effects can also be shown graphically. In Figure S3 we plot the standardized Schoenfeld residuals against time for the variables of interests, namely the real wage and the net-parity fixed effects for the whole period 1540-1850. The line that fits the observations has virtually a zero slope, indicating that the real wage and the parity-fixed effects satisfy the assumption of proportional hazards.

Figure S3: Testing the proportionality assumption

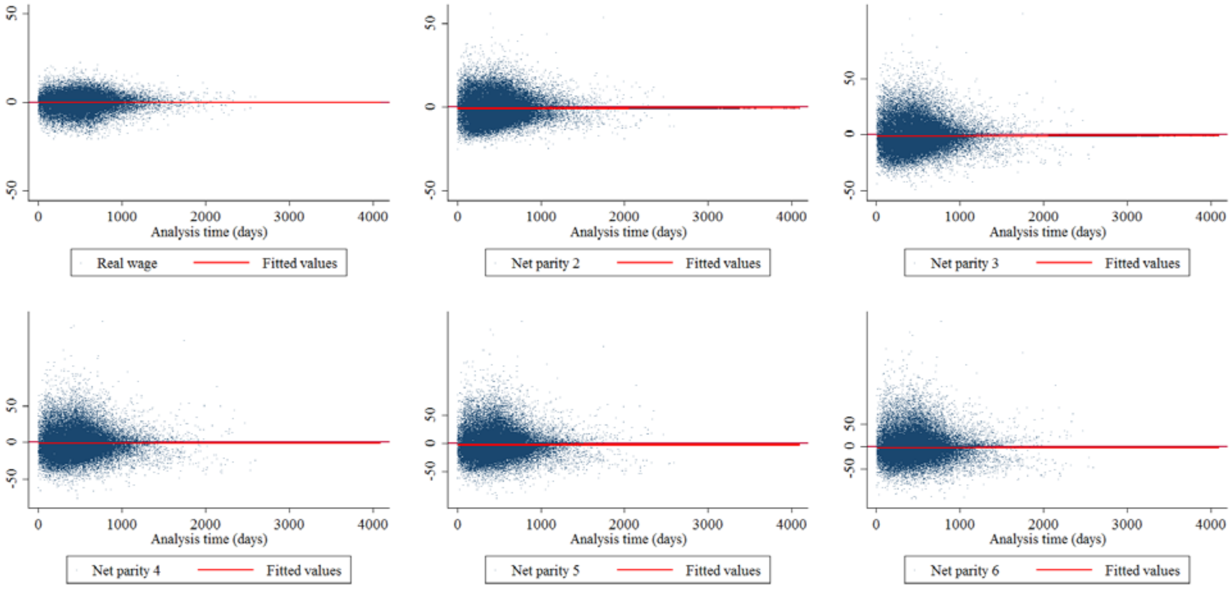

Supplement: Supplementary file 1 — (PDF 303 kb) [file 13524_2017_556_MOESM1_ESM.pdf]
